# The *CADM2* Gene and Behavior: A Phenome-Wide Scan in UK-Biobank

**DOI:** 10.1007/s10519-022-10109-8

**Published:** 2022-07-22

**Authors:** Joëlle A. Pasman, Zeli Chen, Dirk J. A. Smit, Jacqueline M. Vink, Michel C. Van Den Oever, Tommy Pattij, Taco J. De Vries, Abdel Abdellaoui, Karin J. H. Verweij

**Affiliations:** 1 Behavioural Science Institute, Radboud University Nijmegen, Nijmegen, The Netherlands; 2 Department of Medical Epidemiology and Biostatistics, Karolinska Institute, Solna, Sweden; 3 Amsterdam Neuroscience, Department of Psychiatry, Amsterdam UMC, University of Amsterdam, Amsterdam, The Netherlands; 4 Amsterdam Neuroscience, Department of Molecular and Cellular Neurobiology, Center for Neurogenomics and Cognitive Research, Vrije Universiteit Amsterdam, Amsterdam, The Netherlands; 5 Amsterdam Neuroscience, Department of Anatomy and Neurosciences, Amsterdam UMC, Amsterdam, The Netherlands

**Keywords:** *CADM2*, Phenome-wide association study, Risk behavior, Candidate gene, Gene-expression

## Abstract

The cell adhesion molecule 2 (*CADM2*) gene has appeared among the top associations in a wide range of genome-wide association studies (GWASs). This study aims to: (1) examine how widespread the role of *CADM2* is in behavioural traits, and (2) investigate trait-specific effects on *CADM2* expression levels across tissues. We conducted a phenome-wide association study in UK Biobank (N = 12,211–453,349) on 242 psycho-behavioral traits, both at the SNP and the gene-level. For comparison, we repeated the analyses for other large (and high LD) genes. We found significant associations between *CADM2* and 50 traits (including cognitive, risk taking, and dietary traits), many more than for the comparison genes. We show that many trait associations are reduced when taking geographical stratification into account. S-Predixcan revealed that *CADM2* expression in brain tissues was significantly associated with many traits; highly significant effects were also observed for lung, mammary, and adipose tissues. In conclusion, this study shows that the role of *CADM2* extends to a wide range of psycho-behavioral traits, suggesting these traits may share a common biological denominator.

**Supplementary Information:**

The online version contains supplementary material available at 10.1007/s10519-022-10109-8.

In the last 15 years, genome-wide association studies (GWASs) have identified tens of thousands of associations between genetic variants and a range of human behavioral and physical traits. One gene that has popped up surprisingly often in behavioral GWASs is the cell adhesion molecule 2 gene (*CADM2*). Common variations (single nucleotide polymorphisms, SNPs) in the *CADM2* gene have been implicated in various traits, including substance use traits (Pasman et al. 2018; Liu et al. 2019) and risk-taking behavior (Strawbridge et al. 2018; Arends et al. 2021), but also in traits associated with personality (Boutwell et al. 2017), cognition and educational attainment (Ibrahim-Verbaas et al. 2016; Lee et al. 2018), reproductive success (Day et al. 2016), autism spectrum disorders (Casey et al. 2012), physical activity (Klimentidis et al. 2018), BMI/obesity (Locke et al. 2015; Morris et al. 2019), and metabolic traits (Morris et al. 2019).

*CADM2* encodes a member of the synaptic cell adhesion molecules (SynCAMs) involved in synaptic organization and signalling, suggesting that alterations in *CADM2* expression affect neuronal connectivity. *CADM2* is expressed more abundantly in brain tissue than in other tissue and in particular in areas important for reward processing and addiction, including the frontal anterior cingulate cortex (Ibrahim-Verbaas et al. 2016), substantia nigra, and insula (Ndiaye et al. 2019). Accordingly, *CADM2* is a gene that warrants further exploration.

In this study we performed a phenome-wide association analysis (PheWAS), in which we tested for associations of *CADM2* (on SNP and gene level) with a comprehensive selection of psycho-behavioral phenotypes as measured in the UK Biobank cohort. Results provide insights about whether the role of *CADM2* is confined to a specific set of traits or is involved in a wider range of phenotypes. This will inform future studies on the function of *CADM2* and the neurobiological underpinnings of different psycho-behavioral traits. An additional advantage is that the multiple testing burden is reduced as compared to genome-wide studies, resulting in higher statistical power.

UK Biobank is a nationwide study in the United Kingdom containing phenotypic and genetic information for up to 500,000 individuals (Bycroft et al. 2018). We analyzed data from 12,211 to 453,349 UK Biobank participants with European ancestry for whom genetic and phenotypic data were available. About half (54.3%) of the sample was female, and mean age was M = 56.8 (range 39–73, SD = 8.0). We extracted the *CADM2* region 250 kb up- and downstream (all HRC best-guess imputed SNPs from bp 84,758,133 to 86,373,579 on 3p12.1, GRCh37/hg19) and selected 4,265 SNPs with missingness rates < 5%, minor allele frequency > 1%, and *p-*value for violation of Hardy–Weinberg equilibrium above 10^–6^ (quality control details are described in ); with the only difference that we included all HRC imputed SNPs, whereas Abdellaoui (2020) only included HapMap3 SNPs).

We selected 242 psychological and behavioral phenotypes, representing 12 categories, with a sample size above N = 10,000 (for binary traits we used effective sample size ${\text{N}}_{{{\text{eff}}}} = {4}/\frac{{1/N_{cases} }}{{1/N_{controls} }}$). To maximize sample size, we used the first available measurement for each individual; if the first instance was not available, we took the second, otherwise the third, etc. In addition, we included eight traits that were derived for recent genetic studies, including seven substance use traits and educational attainment in years (for an overview of all included traits, see Table S1). Continuous phenotypes were cleaned such that theoretically implausible values were set on missing and extreme values of more than 4 SDs from the mean were winsorized at 4SDs from the mean. Binary and ordinal variables were left unchanged. Ordinal variables were analyzed as continuous variables.

The SNP-based association analyses were performed in fastGWA (Jiang et al. 2019), taking into account genetic relatedness. Analyses were controlled for effects of age, sex, and 25 genetic principal components [PCs, to control for genetic ancestry (Abdellaoui et al. 2019)]. We used linear mixed modeling for all traits and Haseman-Elston regression to estimate the genetic variance component. To test the significance of *CADM2-*associations on gene-level, we conducted a MAGMA gene-based test (de Leeuw et al. 2015), which aggregates the SNP effects (regardless of direction) in a single test of association. We used the default SNP-wise mean procedure (averaging SNP effects across the gene) and checked the results of the SNP-wise top procedure for comparison (this procedure is more sensitive when only a small proportion of SNPs has an effect). As significance threshold for the SNP-based test we adopted a genome-wide significance threshold of *p* < 5E−08. As this is rather stringent given that we test within a single gene, we also used a significance threshold of 0.05 corrected for the number of independent SNPs (n = 133, at R^2^ = 0.10 and 250 kb) and the number of traits, resulting in 0.05/(133*242) = 1.55E−06. For the gene-based test we used a threshold of 2.62E−05, corresponding to 0.05 divided by the total number of genes included in the test (19,082). To provide an estimation of the effect size of the top-SNP for each trait, we used $R^{2} = \frac{{2\beta^{2} MAF\left( {1 - MAF} \right)}}{{2\beta^{2} MAF\left( {1 - MAF} \right) + \left( {se\left( \beta \right)} \right)^{2} 2N MAF\left( {1 - MAF} \right)}}$, as described in (Shim et al. 2015), with adaptations for binary traits as described in (Pasman et al. 2018).

At the SNP-level, 37 traits (out of 242) reached significant associations at a genome-wide corrected *p*-value, and 58 traits at the lenient threshold of *p* < 1.55E−06 (Fig. 1a, Table 1). In the gene-based test, 50 traits showed significant associations (Fig. 1b, Table 1). Thirteen of the 60 substance use traits showed a significant association with *CADM2.* Furthermore, strong associations were found for cognitive ability, risk taking, diet, BMI, daytime sleeping, sedentary behaviors, nervousness-like traits, and reproductive traits. There were fewer associations with occupational, traumatic experiences, social connection, and non-worry related depression traits. Full SNP and gene-based results are provided in Tables S2 and S3a and Figs. S1a and S1b. Table S3b shows the gene-based results for the SNP-wise top procedure. There were some differences with the SNP-wise mean results, with only 34 significant associations and a correlation of r = 0.64 between the *p-*values from the respective tests.

**Fig. 1:**
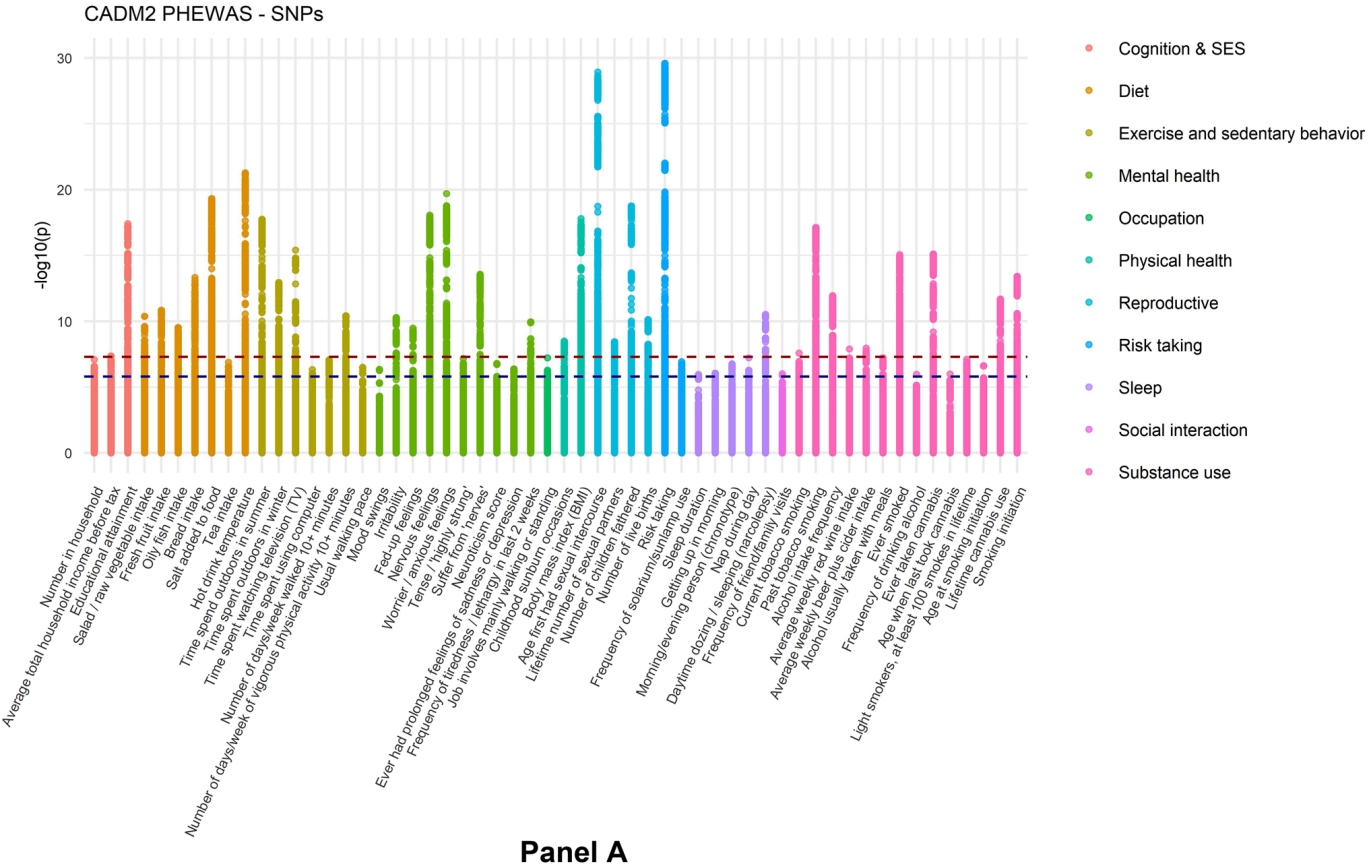

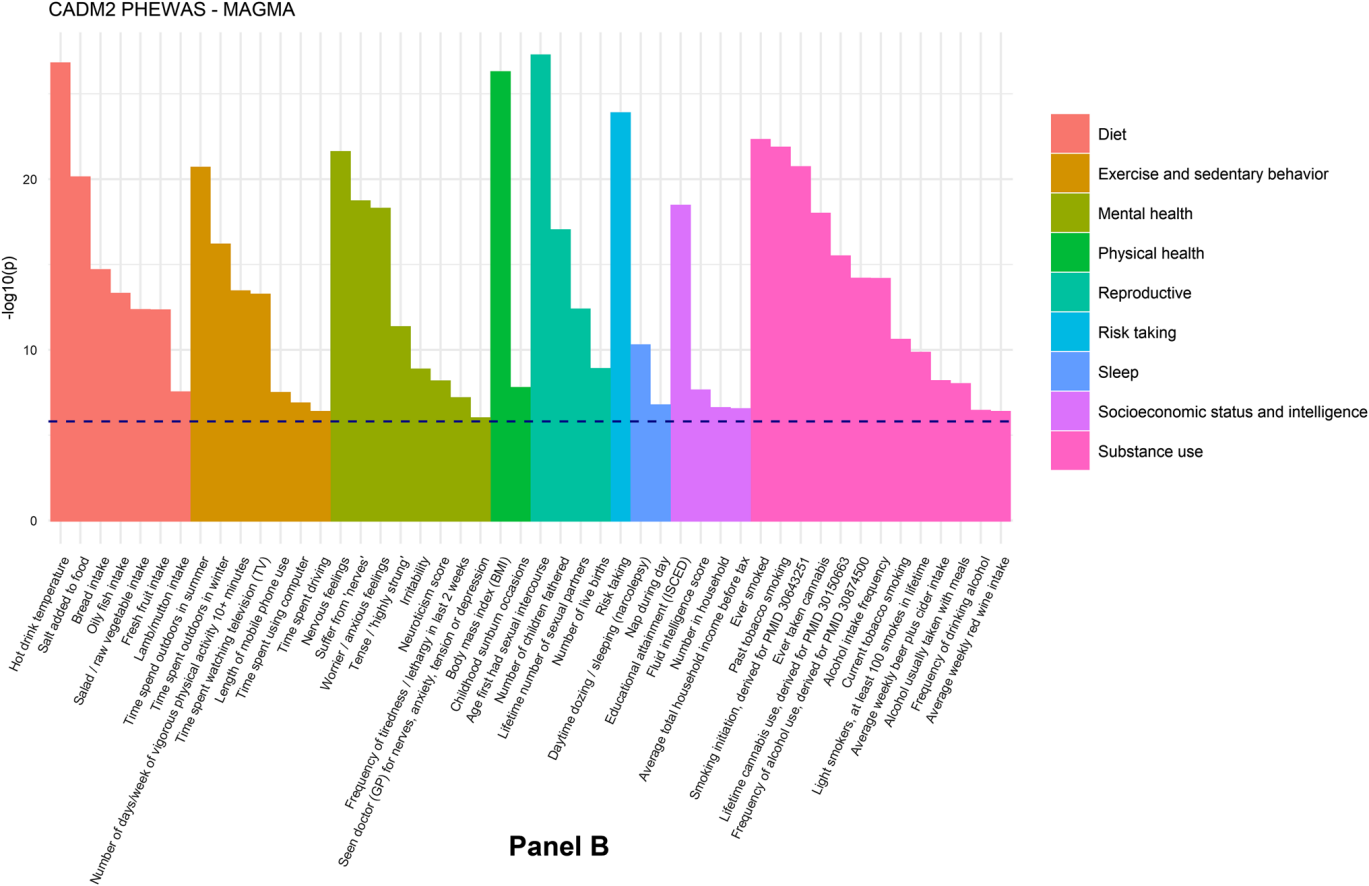
PheWAS results. Panel **A** shows the subset of significant associations of the SNP-based test (58 out of 242 traits). The x-axis shows the traits (colored by trait category) and the y-axis the *p-*values of the association. Each dot represents a SNP association. SNPs exceeding the red horizontal line have a *p-*value significant at a genome-wide threshold of *p* = 5E−08. The blue horizontal line represents the suggestive threshold of *p* = 1.55E−06. Full SNP-based results are given in Supplementary Fig. 1. Panel **B** shows the subset of significant results of the MAGMA gene-based test (50 out of 242 traits), with *p*-values on the y-axis. The red dotted line represents a threshold of *p* = 2.62E−06. The full gene-based results are depicted in Supplementary Fig. S2

**Table 1:** Phenotypes with a significant association with *CADM2* according to the MAGMA gene-based test (SNP-wise mean) at *p* < 2.62E−06

Category	Variable label	N	*p* (Gene)	Top SNP	A1	β	*p*	R^2^ (%)
Diet	Bread intake	448,094	2.23E−15	rs2326128	A	0.016	**4.71E**−**14**	0.013
Diet	Fresh fruit intake	451,780	5.02E−13	rs12638798	T	0.015	**1.43E**−**11**	0.010
Diet	Hot drink temperature	448,694	1.70E−27	rs17023019	A	− 0.021	**5.05E**−**22**	0.021
Diet	Lamb/mutton intake	450,800	3.19E−08	rs10865611	G	0.010	3.19E−06	0.005
Diet	Oily fish intake	451,232	5.37E−14	rs11712915	C	0.016	**2.92E**−**10**	0.009
Diet	Salad/raw vegetable intake	447,874	4.77E−13	rs1248825	A	− 0.015	**4.19E**−**11**	0.010
Diet	Salt added to food	453,342	7.97E−21	rs6780346	C	0.020	**4.77E**−**20**	0.019
Exercise and sedentary behavior	Length of mobile phone use	447,844	3.41E−08	rs13092059	A	0.017	2.36E−06	0.005
Exercise and sedentary behavior	Number of days/week of vigorous physical activity 10 + minutes	431,710	3.91E−14	rs2326123	T	− 0.015	**3.78E**−**11**	0.010
Exercise and sedentary behavior	Time spent driving	446,785	4.51E−07	rs7609594	G	0.010	3.37E−06	0.005
Exercise and sedentary behavior	Time spent outdoors in summer	428,237	2.22E−21	rs62250754	G	0.019	**1.75E**−**18**	0.018
Exercise and sedentary behavior	Time spent outdoors in winter	428,219	6.86E−17	rs62252461	A	− 0.016	**1.10E**−**13**	0.013
Exercise and sedentary behavior	Time spent using computer	449,808	1.42E−07	rs7642644	C	− 0.015	**4.69E**−**07**	0.006
Exercise and sedentary behavior	Time spent watching television (TV)	449,932	5.92E−14	rs9824301	C	− 0.018	**3.93E**−**16**	0.015
Exercise and sedentary behavior	Usual walking pace	450,739	2.03E−06	rs2290338	T	− 0.013	**5.80E**−**07**	0.006
Mental health	Frequency of tiredness/lethargy in last 2 weeks	440,095	6.95E−08	rs818215	C	− 0.014	**1.09E**−**10**	0.010
Mental health	Irritability	433,481	1.46E−09	rs6800177	T	0.016	**5.07E**−**11**	0.001
Mental health	Nervous feelings	441,735	2.61E−22	rs1449386	T	− 0.019	**8.56E**−**19**	0.002
Mental health	Neuroticism score	367,274	7.35E−09	rs818219	C	− 0.012	**1.57E**−**07**	0.008
Mental health	Seen doctor (GP) for nerves, anxiety, tension or depression	450,401	1.03E−06	rs12631564	A	0.010	2.72E−06	0.000
Mental health	Suffer from 'nerves'	436,976	2.07E−19	rs7652808	T	− 0.017	**2.68E**−**14**	0.001
Mental health	Tense/'highly strung'	439,320	4.72E−12	rs9811546	A	− 0.012	**6.58E**−**08**	0.000
Mental health	Worrier/anxious feelings	441,798	5.63E−19	rs62250713	A	− 0.020	**1.93E**−**20**	0.002
Physical health	Body mass index (BMI)	452,169	5.52E−27	rs114781816	A	0.024	5.46E−04	0.003
Physical health	Childhood sunburn occasions	339,522	1.77E−08	rs9880919	A	0.017	**3.16E**−**09**	0.010
Reproductive	Age first had sexual intercourse	398,273	5.84E−28	rs62263912	G	− 0.027	**1.15E**−**29**	0.033
Reproductive	Lifetime number of sexual partners	371,577	4.45E−13	rs4856598	A	0.014	**3.40E**−**09**	0.010
Reproductive	Number of children fathered	205,643	1.02E−17	rs1368750	T	0.029	**1.67E**−**19**	0.039
Reproductive	Number of live births	245,754	1.34E−09	rs1972994	A	0.020	**7.43E**−**11**	0.017
Risk taking	Risk taking	437,506	1.42E−24	rs7649296	A	0.016	4.97E−05	0.001
Sleep	Daytime dozing/sleeping (narcolepsy)	451,752	5.63E−11	rs960986	T	− 0.015	**2.95E**−**11**	0.010
Sleep	Nap during day	453,172	1.81E−07	rs3943782	G	0.012	**5.85E**−**08**	0.007
Social interaction	Frequency of friend/family visits	450,658	1.56E−06	rs1248860	G	0.010	**9.34E**−**07**	0.005
Socioeconomic status and intelligence	Average total household income before tax	390,130	3.05E−07	rs426444	T	− 0.013	**4.34E**−**08**	0.008
Socioeconomic status and intelligence	Educational attainment transformed to ISCED categories, derived for PMID 27225129	449,507	3.76E−19	rs11915747	G	0.019	**3.83E**−**18**	0.017
Socioeconomic status and intelligence	Fluid intelligence score	233,219	2.42E−08	rs72903244	A	− 0.047	**2.84E**−**08**	0.013
Socioeconomic status and intelligence	Number in household	450,766	2.67E−07	rs62250661	A	− 0.011	**7.80E**−**08**	0.006
Substance use	Alcohol intake frequency	453,062	7.17E−15	rs9814516	T	− 0.017	**1.10E**−**12**	0.011
Substance use	Alcohol usually taken with meals	231,191	1.03E−08	rs12493621	C	0.016	**5.74E**−**08**	0.001
Substance use	Average weekly beer plus cider intake	322,313	7.02E−09	rs9824301	C	− 0.013	**1.09E**−**08**	0.010
Substance use	Average weekly red wine intake	321,719	4.40E−07	rs382210	G	− 0.015	**1.28E-08**	0.010
Substance use	Current tobacco smoking	453,148	2.65E−11	rs56262138	A	− 0.013	**2.61E**−**08**	0.000
Substance use	Ever smoked	451,812	5.16E−23	rs6790699	A	0.017	**8.26E**−**16**	0.001
Substance use	Ever taken cannabis	146,758	1.08E−18	rs62263912	G	0.031	**7.61E**−**16**	0.013
Substance use	Frequency of alcohol use, derived for PMID 30874500	453,070	7.02E−15	rs9814516	T	− 0.017	**1.09E**−**12**	0.011
Substance use	Frequency of drinking alcohol	146,785	3.78E−07	rs9832119	T	− 0.019	**1.07E**−**06**	0.016
Substance use	Lifetime cannabis use, derived for PMID 30150663	146,758	3.41E−16	rs67336646	T	0.026	**2.02E**−**12**	0.007
Substance use	Light smokers, at least 100 smokes in lifetime	121,322	1.53E−10	rs62253088	T	0.023	**7.45E**−**08**	0.004
Substance use	Past tobacco smoking	416,587	1.45E−22	rs6780346	C	− 0.019	**7.48E**−**18**	0.002
Substance use	Smoking initiation, derived for PMID 30643251	301,588	2.08E−21	rs62263910	G	0.020	**3.82E**−**14**	0.000

The top-SNP for the phenotype is given with the minor allele (A1), beta (β), *p*-value (*p*), and percentage of explained variance in the respective trait [R^2^ (%)]. Most top-SNPs were significant at *p* < 1.55E−6 (bold-faced)

For binary traits, the effective sample size is given (determined using ${\text{N}}_{{{\text{eff}}}} = {4}/\frac{{1/N_{cases} }}{{1/N_{controls} }}$)

In the main PheWas analysis, we controlled for potential bias in estimated associations due to population stratification using 25 genetic PCs. However, *CADM2* is located in a long-range linkage disequilibrium (LD) region, making it potentially unfeasible to adequately control for population structure with PCs. Also, there may be genetic signal picked up by genetic association analyses that is due to social stratification, which will not be accounted for by these 25 PCs. We therefore performed a sensitivity analysis in which we—in addition to the 25 PCs—controlled for the participants’ region of birth and region of current address (see Supplementary methods). Controlling for these geographical covariates attenuated the association results: from the 50 significant trait associations at the gene level, 26 were no longer significant, and on average the betas of the top-SNPs within these genes were attenuated with by 16% (Table S3c, Fig. S1c). These findings implicate that (social) stratification introduces regional-level gene-environment correlations that affect the genetic association results (Abdellaoui 2020), although the lower number of significant gene associations could in part be the result of reduced power due to the inclusion of hundreds of dummy covariates coding geographical region. Even after controlling for effects of stratification/gene-environment correlation there remained evidence of widespread associations with *CADM2*.

We assessed whether the high number of associations discovered for *CADM2* was unusual or similar to those found for other genes. We therefore selected a random set of 50 genes (that were maximum 50% smaller or larger), repeated the SNP-based analysis for these genes and compared the number of traits with significant associations. Most of the random comparison genes contained fewer than 5 SNP-trait associations, with an average of 2.6 associated traits per gene and a maximum of 13 (as compared to 50 for *CADM2*; Table S4). We additionally made a comparison with five large genes from regions with a similar level of LD as the *CADM2* region (five was the number of similarly sized genes that were within LD regions defined in Price et al. (2008)). The number of significant associations within these genes was still substantially lower than those in *CADM2* (maximum 6, Table S5). Results from these comparison analyses show that the high number of associations discovered for *CADM2* is exceptional (Fig. S2).

The *CADM2* SNPs that showed the highest number of significant trait-associations (with a maximum of 26 traits at *p* < 1.55E−6, Table S6) clustered around loci at 85.53 and 85.62 Mb. As can be seen in Fig. 2, most SNPs that were independently (LD R^2^ < 0.01, distance > 250 kb) significantly associated with at least one trait cluster in the middle of the gene, a region rich in expression quantitative trait loci (eQTLs).

**Fig. 2:**
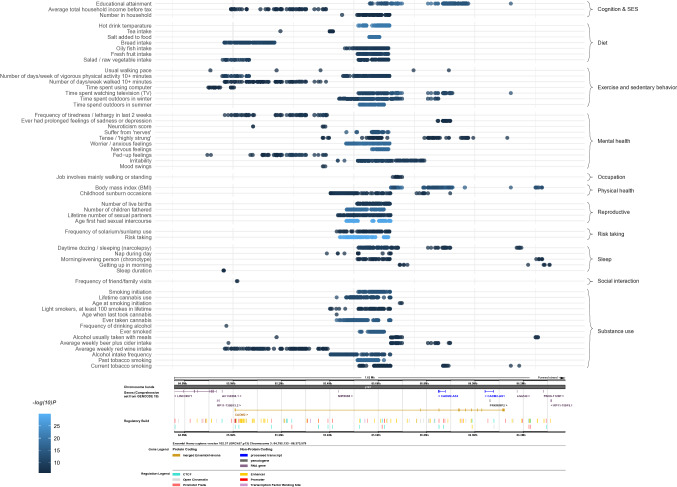
The top 100 most significant SNPs for each trait with at least 1 significant SNP. The x-axis represents the base pair position, and the panel below shows information on the *CADM2* transcripts as derived from https://www.ensembl.org/

To further investigate eQTL effects, we used S-Predixcan with the 49 precalculated GTEx Elastic Net models (Barbeira et al. 2018) to establish association between traits and *CADM2* expression levels in 17 brain and non-brain tissues (see Supplementary Methods). From each trait category (with significant associations, N = 9) we selected the trait with the strongest association with *CADM2*. For all traits we found significant associations with *CADM2* expression in multiple tissues (Table S7, Fig. 3). Highly significant effects were observed for lung, mammary, and adipose tissues across all traits. *CADM2* expression in brain tissues was significantly associated with many traits, including risk taking, nervous feelings, and hot drink temperature. Smaller to negligible effects were observed for spleen and tibial nerve tissues.

**Fig. 3:**
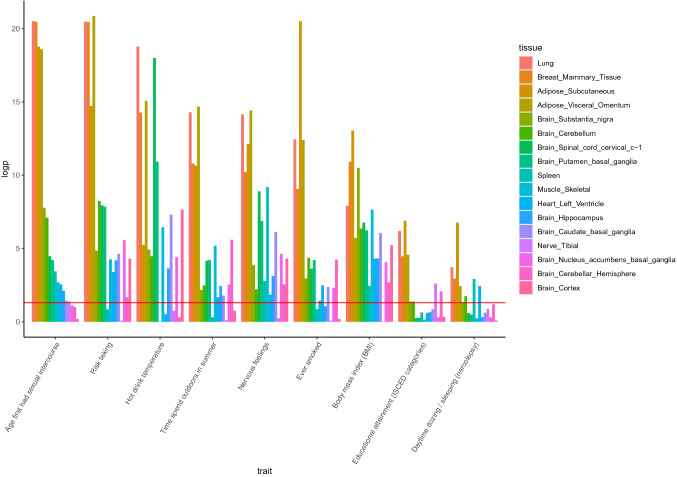
S-predixcan results testing association between the GWASs of selected top traits and *CADM2* expression in a range of tissues. S-PrediXcan was run with elastic net models based on GTEx v8 expression data. On the y-axis are the FDR-corrected log-transformed p-value, with the red line representing the significance threshold of *p*_FDR_ = 0.05

This PheWAS showed that *CADM2* was involved in a wide spectrum of traits, thereby reproducing and extending on previous findings. Interestingly, comparison with 50 other genes showed that this number of trait-associations was exceptionally high, emphasizing the distinctive role of *CADM2* in psycho-behavioral traits. Substance use traits did not seem highly overrepresented among the significantly associated traits, suggesting that the involvement of *CADM2* is of a more general nature. Many of the associations we found have been reported in previous literature [Table S6, based on GWAS Catalog (Buniello et al. 2019)]. Others were previously calculated by Neale et al. and Watanabe et al. using PheWAS in the same dataset, but not reported in a scientific paper [see Open Targets Genetics Platform, Carvalho-Silva et al. (2019), or GWAS Atlas, Watanabe et al. (2019)]. We add to these findings by identifying trait associations that remain strong after taking into account geographical stratification (e.g., age at first sexual intercourse, nervous feelings, and risk taking), and how the strongest traits were associated with differential *CADM2* expression. The variance explained by *CADM2* was highest for number of children fathered, age at first sexual intercourse, and hot drink temperature. Overall, effect sizes were small (less than 0.04% for number of children), in range with what is normally found for single variants. Few associations were found in the social interaction, sleep, traumatic experiences, and occupational categories. Also, there were not many mental health traits that showed an association (8 out of 52 traits). It is interesting to note the significant associations with worry and nervousness-like traits in the absence of association with other depression- and anxiety-related traits. There may be something specific to these seemingly overlapping traits, translating to distinct biological pathways.

It needs to be noted that sample sizes for the phenotypes differed substantially (from N = 12,211 to 453,349), and as such, it is possible that the pattern of associations was driven in part by differences in power. The correlation between sample size and *p*-value of the gene-based test was moderate and significant, r = − 0.38 (*p* = 1.42E−9) showing that well-powered traits were more likely to result in a significant association. It is clear that high power was a requirement: the effect sizes of *CADM2* were diminutive, as is expected for single genes and complex traits. Also, our tests were limited to the psycho-behavioral traits measured in the UK-Biobank; inclusion of more measures, such as longitudinal or non-self-report measures could contribute to a more complete picture. Still, the range of tested traits was quite broad and enabled us to discern interesting patterns.

More research is needed to elucidate these links between *CADM2* and this spectrum of psycho-behavioral traits in terms of neurobiological mechanisms. For example, it could be that *CADM2* is important for the learning aspects of behavior, given its role in synaptic connectivity. Speculatively, *CADM2* could then contribute to reward-learning and associative learning, giving rise to risky behavior, substance use, and other kinds of behaviors that involve such processes (Volkow et al. 2016).

This study presents a comprehensive and rigorous test of associations between *CADM2* and psycho-behavioral traits, showing strong associations for a wide range of traits. Results could be used as starting point for future research into the function of *CADM2.* Research on the trait-associations and function of *CADM2* will further our understanding of the biology of behavior.

## Supplementary Information

Below is the link to the electronic supplementary material.

Supplementary file1 (PDF 5824 KB)

Supplementary file2 (DOCX 31 KB)

Supplementary file3 (XLSX 21125 KB)

## Data Availability

Data from the UK-Biobank are available upon application.

## Code Availability

Codes and scripts used for the current project can be requested from the authors.
