# Supplementary material for: The CADM2 Gene and Behavior: A Phenome-Wide Scan in UK-Biobank
Source: Behav Genet. 2022 Jul 22;52(4-5):306–14. doi: 10.1007/s10519-022-10109-8 (PMC9463269; doi:10.1007/s10519-022-10109-8)
Supplement: Supplementary file 1 — Supplementary file1 (PDF 5824 KB) [file 10519_2022_10109_MOESM1_ESM.pdf]

Supplementary Figures

Figure S1. PheWAS results. Panel **a)** shows the associations of the SNP-based test for all 242 traits. The x-axis shows the traits (colored by trait category) and the y-axis the p-values of the association. Each dot represents a SNP association. SNPs exceeding the red horizontal line have a p-value significant at a genome-wide threshold of p=5E-08. The blue horizontal line represents the suggestive threshold of p=1.55E-06. Full SNP-based results are given in Supplementary Figure 1. Panel **b)** shows the results of the MAGMA gene-based test (for all 242 traits), with p-values on the y-axis. The red dotted line represents a threshold of p=2.62E-06. Panel **c)** shows the results of the MAGMA gene-based test (for all 242 traits), after additional correction for MSOA regions (birthplace and current address). p-values on the y-axis. The red dotted line represents a threshold of p=2.62E-06.

Figure S2. Number of significant trait associations per comparison gene (with SNP associations at p<5E-08). On the right the CADM2 gene, the only one with more than 13 trait associations. See Tables S4 and S5 for the association results per comparison gene.

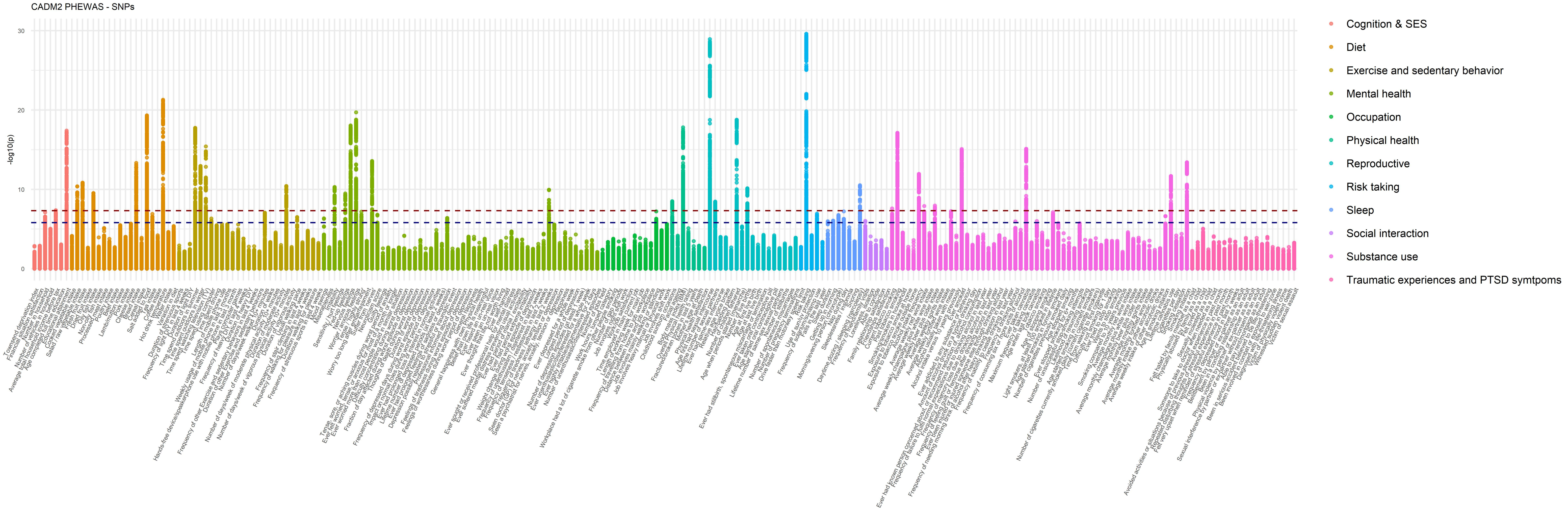
CADM2 PHEWAS - SNPs
-log10(p)
30
20
10
0
Cognition & SES
Diet
Exercise and sedentary behavior
Mental health
Occupation
Physical health
Reproductive
Risk taking
Sleep
Social interaction
Substance use
Traumatic experiences and PTSD symtpoms

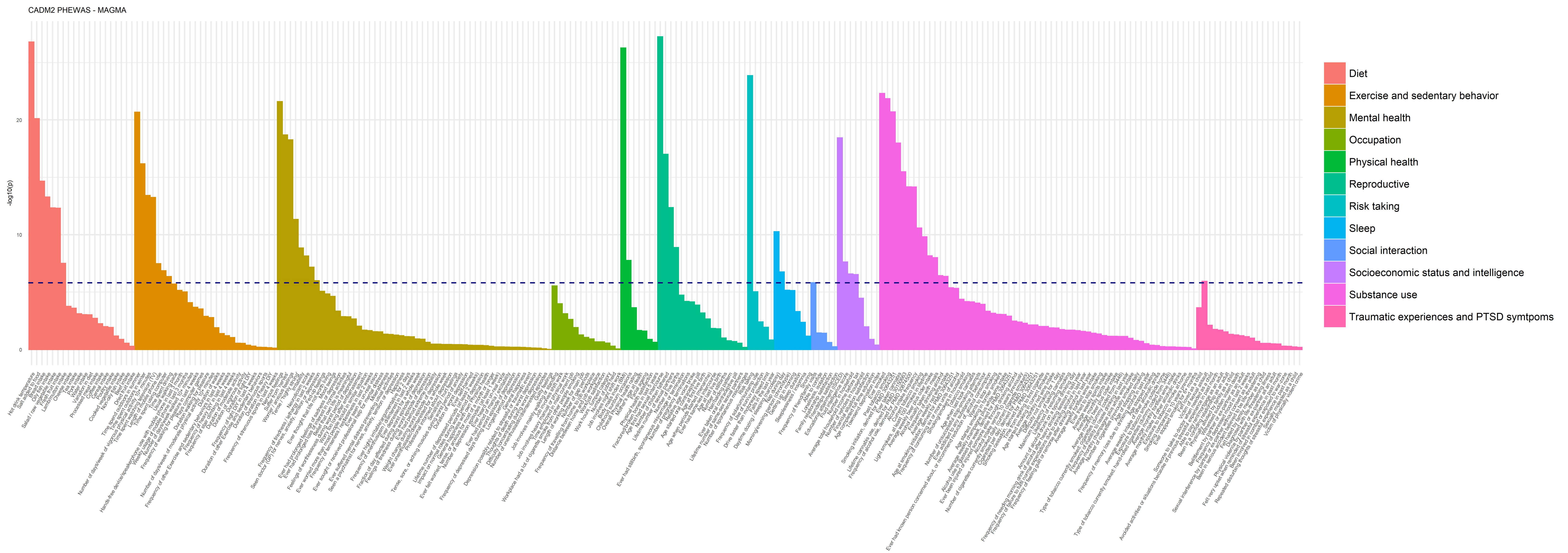
CADM2 PHEWAS - MAGMA
-log10(p)
0
10
20
Diet
Exercise and sedentary behavior
Mental health
Occupation
Physical health
Reproductive
Risk taking
Sleep
Social interaction
Socioeconomic status and intelligence
Substance use
Traumatic experiences and PTSD symtpoms

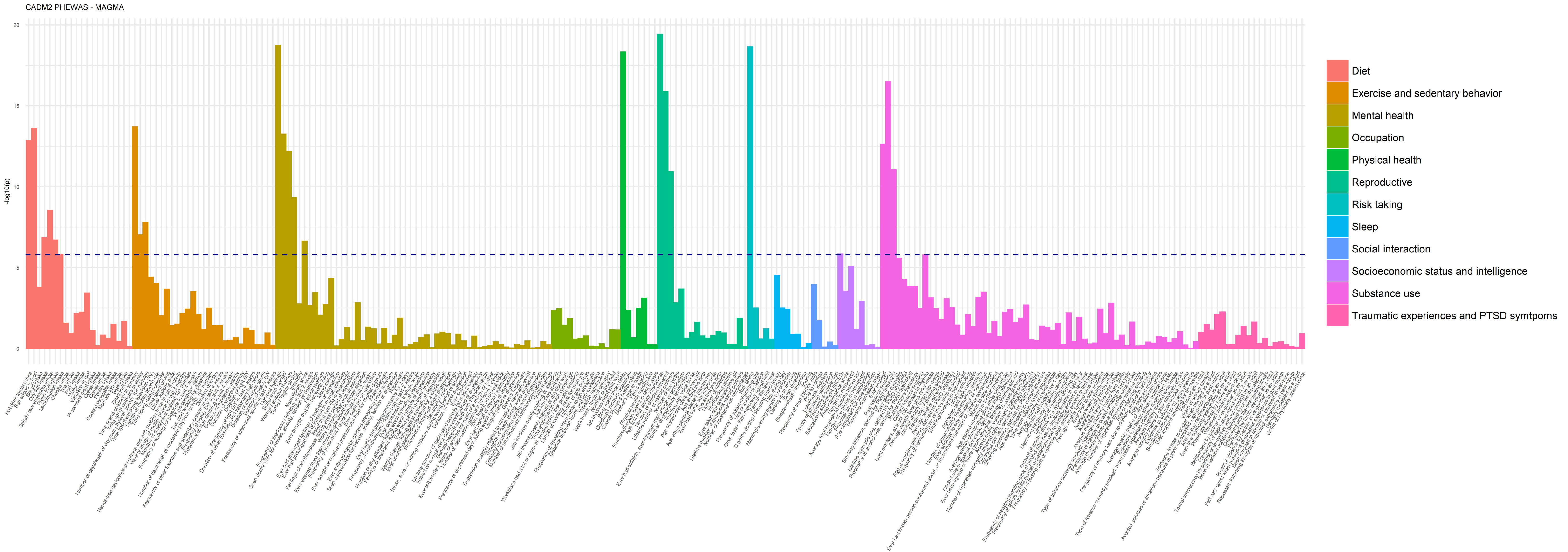
CADM2 PHEWAS - MAGMA
-log10(p)
20
15
10
5
0
Diet
Exercise and sedentary behavior
Mental health
Occupation
Physical health
Reproductive
Risk taking
Sleep
Social interaction
Socioeconomic status and intelligence
Substance use
Traumatic experiences and PTSD symtpoms

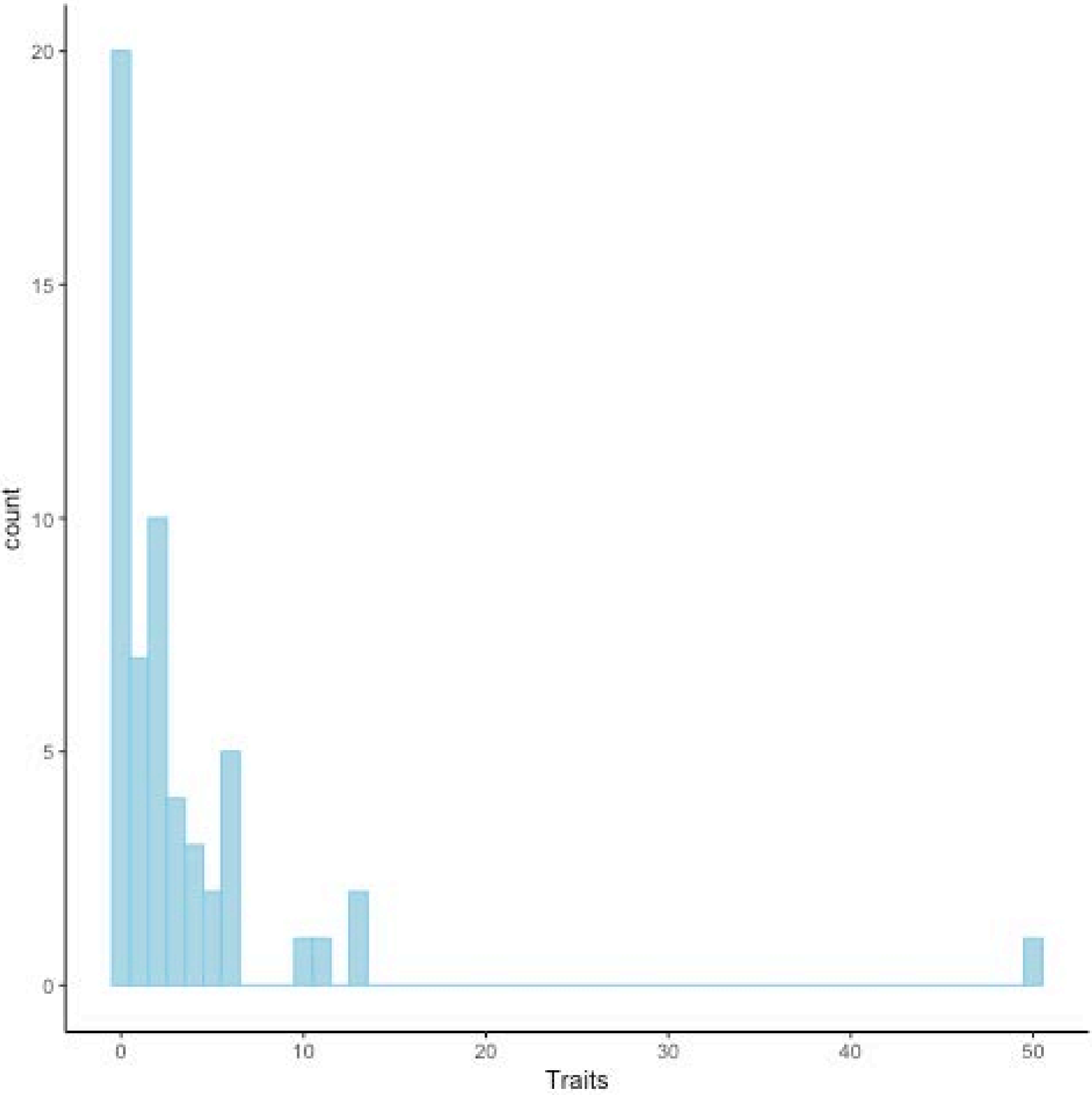

20
15
10
5
0
count
0
10
20
30
40
50
Traits
