# Supplementary material for: The CADM2 Gene and Behavior: A Phenome-Wide Scan in UK-Biobank
Source: Behav Genet. 2022 Jul 22;52(4-5):306–14. doi: 10.1007/s10519-022-10109-8 (PMC9463269; doi:10.1007/s10519-022-10109-8)
Supplement: Supplementary file 2 — Supplementary file2 (DOCX 31 KB) [file 10519_2022_10109_MOESM2_ESM.docx]

# The *CADM2* gene and behavior: A phenome-wide scan in UK-Biobank

Joëlle A. Pasman, Zeli Chen, Dirk J.A. Smit, Jacqueline M. Vink, Michel C. Van Den Oever, Tommy Pattij, Taco J. De Vries, Abdel Abdellaoui, & Karin J.H. Verweij

**Supplementary Methods**

*Controlling for geographic regions*

The geographic regions were obtained by mapping latitude and longitude coordinates of the birthplace and current address to local authority areas or Middle Layer Super Output Area (MSOA) regions. MSOA regions are defined as a set of adjacent output areas designed to have comparable population sizes and to be: “as socially homogeneous as possible based on tenure of household and dwelling type” (Office for National Statistics 2020). Birthplace and current address locations were based on the coordinates provided by fields 129 (latitude) and 130 (longitude) for birthplace, and fields 22702 (longitude) and 22703 (latitude) for current address). The participants’ coordinates were rounded to 1 km, and were then mapped to the nearest MSOA region using a shape file obtained from the InFuse website, which is part of the UK Data Service Census Support (Office for National Statistics 2011). The R-packages sp (v1.4-4) and rgdal (v1.5-18) were used to merge the spatial data from the MSOA shapefile (Bivand et al. 2008; Pebesma and Bivand 2005). We used these MSOA regions to rerun the SNP and gene-based test of associations controlling for geographical region, by adding dummy variables based on birthplace and dummy variables based on current address. We only included regions with ≥ 100 UK Biobank participants. This resulted in in **816** regions for the birthplace and **1,747** regions for the current address. For more information on this method, please see Abdellaoui et al. (2021).

*S-Predixcan Imputed Expression Analysis*

To examine the extent to which the traits were associated with *CADM2* expression levels in specific adult tissues (both in the brain and other tissues), we performed S-Predixcan analysis on the genetic association results. S-Predixcan is based on Predixcan (Gamazon et al. 2015) which uses RNAseq expression and SNP information to build sparse elastic net models. Here we used the 49 precalculated elastic net models based on the GTEx v8 database (Aguet et al. 2020), which consists of one model for *CADM2* for each of the tissues. Note that only for 17 tissues there was a valid elastic net prediction model.

In Predixcan, these models are used to impute expression for a gene (here *CADM2)* in a specific tissue based on each individual’s genetic profile (SNP dosage). These imputed gene-expressions are then associated with the phenotype, resulting in tissue specific associations of gene-expression with the phenotype. S-Predixcan (Barbeira et al. 2018) is an extension of Predixcan and can be used with summary statistics. Given the strong correlations between the various psycho-behavioural traits, we selected the trait with the strongest association with *CADM2* from each trait category with significant associations (N=9). Significance testing was performed using FDR corrected p-values (across all trait/tissue combinations).

**References**

Office for National Statistics (2020) Census Geography. <https://www.ons.gov.uk/methodology/geography/ukgeographies/censusgeography>,

Office for National Statistics (2011) Census aggregate data. UK Data Service, <https://infuse.ukdataservice.ac.uk/>,

Bivand RS, Pebesma EJ, Gómez-Rubio V, Pebesma EJ (2008) Applied spatial data analysis with R. Springer

Pebesma E, Bivand RS (2005) S classes and methods for spatial data: the sp package. R news 5(2):9-13

Abdellaoui A, Verweij KJH, Nivard MG (2021) Geographic Confounding in Genome-Wide Association Studies. bioRxiv 2021.2003.2018.435971

Gamazon ER, Wheeler HE, Shah KP, Mozaffari SV, Aquino-Michaels K, Carroll RJ, . . . Consortium GT (2015) A gene-based association method for mapping traits using reference transcriptome data. Nat Genet 47(9):1091-1098

Aguet F, Anand S, Ardlie KG, Gabriel S, Getz GA, Graubert A, . . . Volpi S (2020) The GTEx Consortium atlas of genetic regulatory effects across human tissues. Science 369(6509):1318-1330

Barbeira AN, Dickinson SP, Bonazzola R, Zheng J, Wheeler HE, Torres JM, . . . Visualization—Ucsc Genomics Institute UoCSC (2018) Exploring the phenotypic consequences of tissue specific gene expression variation inferred from GWAS summary statistics. Nature Communications 9(1):1825
